# People’s Perception of Well-Being during the COVID-19 Pandemic: A Case Study in Japan

**DOI:** 10.3390/ijerph191912146

**Published:** 2022-09-25

**Authors:** Daisuke Sasaki, Anawat Suppasri, Haruka Tsukuda, David N. Nguyen, Yasuaki Onoda, Fumihiko Imamura

**Affiliations:** 1 International Research Institute of Disaster Science (IRIDeS), Tohoku University, Sendai 980-8572, Japan; 2 Department of Architecture and Building Science, School of Engineering, Tohoku University, Sendai 980-8579, Japan

**Keywords:** COVID-19, disaster science, evidence-based policymaking, ordinal logistic regression, principal component analysis, compound indicator, single-person households, Japan

## Abstract

This study aims to examine people’s perception of well-being during the COVID-19 pandemic in Japan and quantitatively clarify key factors towards realizing evidence-based policymaking. In March 2022, 400 participants responded to a survey conducted through Rakuten Insight. The authors applied an ordinal logistic regression (OLR), followed by principal component analysis (PCA), to create a new compound indicator (CI) to represent people’s perception of well-being during the pandemic in addition to ordinary least squares (OLS) regression with a forward-backward stepwise selection method, where the dependent variable is the principal component score of the first principal component (PC1), while the independent variables are the same as the abovementioned OLR. Consequently, while analyzing OLR, some independent variables showed statistical significance, while the CI provided an option to grasp people’s perception of well-being. Furthermore, family structure was statistically significant in all cases of OLR and OLS. Moreover, in terms of the standardized coefficients (beta) of OLS, the family structure had the greatest impact on the CI. Based on the study results, the authors advocate that the Japanese government should pay more attention to single-person households affected by the COVID-19 pandemic.

## 1. Introduction

Coronavirus disease (COVID-19), an infectious disease caused by the SARS-CoV-2 virus, was first detected in late 2019 and rapidly spread to the rest of the world in 2020, leading the World Health Organization (WHO) to declare the outbreak as a global pandemic in March 2020 [1]. Researchers have explored the psychological, social, and neuroscientific effects of COVID-19 and presented longer-term strategies for mental health science research [2]. Along with the approach of mental health science research, some researchers have investigated COVID-19 and its implications from a resilience point of view and made some recommendations for the sake of disaster risk reduction [3,4,5]. In terms of the well-being of people during the COVID-19 pandemic, although we can find abundant literature focusing on how well-being has been affected by COVID-19 mainly in Europe, the US, and China, there is a lack of literature conducting a case study in Japan [6].

The existing literature regarding well-being is as follows. Saladino et al. [7] highlighted the impact on the psychological well-being of the groups most exposed to COVID-19, such as children, college students, and health workers. Sibley et al. [8] focused on the effects of the COVID-19 pandemic and nationwide lockdown on trust, attitudes toward government, and well-being. Brodeur et al. [9] utilized Google Trends data to examine whether COVID-19 and the associated lockdowns implemented in Europe and the United States led to changes in search terms related to the topic of well-being. Lesser and Nienhuis [10] assessed how preemptive measures, such as social distancing and closure of municipal and provincial recreation facilities, impacted physical activity behavior and the well-being of Canadians; they suggested that health-promoting measures directed towards inactive individuals may be essential to improving well-being. Nienhuis and Lesser [11] have also assessed whether sex differences exist in physical activity and well-being since COVID-19 and explored how barriers or facilitators to physical activity may explain these differences. Dahlen et al. [12] demonstrated a positive and robust association between changes in daily activity levels and corresponding changes in psychological well-being. Feitelson et al. [13] assessed the well-being effects of COVID-19 in Israel by analyzing the pandemic’s impact on several well-being indicators. Other studies have highlighted the psychological well-being of parents and their children. For example, Gassman-Pines et al. [14] examined the hypothesis that the crisis had worsened the psychological well-being of parents and children using daily survey data collected before and after the crisis started. Patrick et al. [15] investigated how the pandemic and mitigation efforts affected the physical and emotional well-being of parents and children in the United States in early June 2020. Huebener et al. [16] suggest that public policy measures taken to contain COVID-19 can have large effects on family well-being, based on a novel representative survey of parental well-being collected between May and June 2020 in Germany.

Furthermore, previous studies have examined the factors that affect well-being during the pandemic era. O’Connor et al. [17] indicate that the mental health and well-being of the UK adult population appear to have been affected during the initial phase of the COVID-19 pandemic; they state that the increasing rates of suicidal thoughts across waves, especially among young adults, are concerning. Coppola et al. [18] suggest that family is a protective factor with respect to mental health because the perceived mental health of those who did not live alone, and especially those who had to take care of small children, appear to be higher due to a seemingly greater ability to activate coping resources. Özmen et al. [19] stated that the scores of the participants in the survey conducted in Turkey in April 2020, regarding the fear of COVID-19, showed statistically significant differences according to the following variables: age, gender, education level, working status, the presence of pre-existing chronic diseases, regular drug use, and income level. Tomaz et al. [20] advocated that a larger social network, more social contact, and better perceived social support seemed to protect against loneliness and poor well-being; thus, addressing loneliness and social support in older adults is of significance. Fingerman et al. [21] also suggest that older adults who live alone may be more reactive to social contact during the COVID-19 outbreak than those who reside with others. In addition to the abovementioned previous studies, we can also find useful findings for evidence-based policymaking (EBPM) in the field of disaster science [22,23,24,25,26,27].

Under such circumstances, Suppasri et al. [28] conducted a survey between 5 and 9 November 2020, with a total of 600 respondents in Japan, based on a survey conducted by the European Commission’s Joint Research Centre (EU-JRC) and University College London (UCL) to facilitate future international comparisons [29]. One of the preliminary results obtained from a simple tabulation showed that the respondents believed that support for basic needs, such as goods and other utilities, should be prioritized, followed by support for low-income persons and support for persons who own their own businesses [28]. However, the influence of the COVID-19 pandemic on people’s perceptions of their well-being in Japan remains an open question. This study is follow-up research, in which the authors developed and conducted a second set of surveys based on Suppasri et al. [28]. Thus, this study aims to examine people’s perception of well-being during the COVID-19 pandemic in Japan and clarify key factors in a quantitative manner toward the realization of EBPM in the future. In this regard, this study could contribute to the literature on the well-being of people in Japan during the COVID-19 pandemic, in that it provides lessons learned from Japanese case.

## 2. Methodology

The questionnaire survey was conducted through Rakuten Insight in March 2022 with a total of 400 respondents. The targeted areas that were selected as follows: Hokkaido (*n* = 72, 18.0%), Iwate Prefecture (*n* = 37, 9.3%), Miyagi Prefecture (*n* = 31, 7.8%), Saitama Prefecture (*n* = 20, 5.0%), Chiba Prefecture (*n* = 9, 2.3%), Tokyo Metropolis (*n* = 38, 9.5%), Kanagawa Prefecture (*n* = 25, 6.3%), Kyoto Prefecture (*n* = 12, 3.0%), Osaka Prefecture (*n* = 43, 10.8%), Hyogo Prefecture (*n* = 33, 8.3%), Fukuoka Prefecture (*n* = 38, 9.5%), Saga Prefecture (*n* = 4, 1.0%), Nagasaki Prefecture (*n* = 10, 2.5%), Kumamoto Prefecture (*n* = 15, 3.8%), Oita Prefecture (*n* = 5, 1.3%), Miyazaki Prefecture (*n* = 3, 0.8%), and Kagoshima Prefecture (*n* = 5, 1.3%). Thus, the targeted areas were Hokkaido, the Tohoku region, the capital area, the Kansai region, and the Kyushu region. There were 218 (54.5%) male and 182 (45.5%) female respondents. The average age was 49.1 years, ranging from 25 to 69 years. In terms of the respondents’ educational level and employment status, 193 (48.3%) graduated from university, 160 (40.0%) graduated from high school, 256 (64.0%) were employed, and 55 (13.8%) were homemakers. All the questions were presented in Japanese. The survey had 59 questions, which included demographic questions, such as gender, age, annual household income, and family structure. The software package used for statistical analysis in this study was SPSS Statistics 28.

First, the authors apply ordinal logistic regression (OLR). OLR is a regression method for ordinal dependent variables that have been used in social data analysis in the existing literature, such as DeMaris [30]. The dependent variables in this study are the four proxies of people’s perception of well-being during the COVID-19 pandemic: (i) change in job satisfaction, (ii) change in satisfaction with family, (iii) change in psychological well-being, and (iv) change in economic well-being. In this study, the authors have chosen the above four variables as the dependent variables while referring to the investigation regarding well-being by the Cabinet Office, Government of Japan [31]. These dependent variables used a 5-point Likert scale ranging from Heavily deteriorated, Deteriorated, Unchanged, Improved, and Heavily improved. The independent variables are the following 22 variables: change in daily food, water, electricity, and heat consumption, change in the use of public transportation, change in use of private transportation, change in use of medical and hospital services, change in use of banking and financial services, change in use of telephone and Internet services, concerns about the lack of economic recovery measures, concerns about the risk of a new wave of COVID-19 infection spreading, concerns about possible disruption of essential and basic services, concerns about the possibility of simultaneous occurrence of natural hazards, concerns about the risk of simultaneous acts of terrorism, cyber-attacks, riots, age, number of households, gender, education level, family structure, length of residency, existence of dependents, existence of pets, employment, annual household income, and residency in the Greater Tokyo Area, which consists of the Tokyo Metropolis, Kanagawa, Chiba, and Saitama Prefectures. The first six variables and the subsequent five variables also utilized 5-point Likert scales ranging from -2 (Heavily decrease) to 2 (Heavily increase) and from 0 (None at all) to 4 (Quite a lot), respectively. Age and the number of household variables are set on a ratio scale. The last nine variables are used as dummy variables. Gender takes a value of 1 (Female) or 0 (Male). Similarly, the education level is 1 (university graduate or above) or 0 (otherwise). The family structure is 1 (single-person household) or 0 (otherwise). The length of residence is 1 (10 years or more) or 0 (otherwise). The existence of dependents is 1 (yes) or 0 (no). The existence of pets is 1 (yes) or 0 (no). Employment was scored as 1 (employed) or 0 (otherwise). The annual household income is 1 (less than five million yen) or 0 (otherwise). Residency in the Greater Tokyo Area is either 1 or 0 (otherwise).

Subsequently, we conducted a principal component analysis (PCA) to create a new compound indicator (CI). The origin of PCA dates back to early 20th-century literature, such as Hotelling [32]. Jolliffe and Cadima [33] explain that PCA is a methodology for reducing the dimensionality of a dataset, which minimizes information loss while increasing interpretability by reducing dimensionality. In particular, PCA creates new uncorrelated variables while maximizing variance by solving an eigenvalue/eigenvector problem. In this study, we adopt the first principal component (PC1) of the four variables of people’s perception of well-being during the COVID-19 pandemic as a new CI; then, we calculate the principal component score (PCS) of PC1. Furthermore, we apply ordinary least squares (OLS) regression with a forward-backward stepwise selection method, where the dependent variable is the PCS of the PC1, while the independent variables are the same as the abovementioned OLR, assuming that the residuals follow a normal distribution.

## 3. Results

### 3.1. Descriptive Statistics of the Dependent Variables

The frequency distributions of the dependent variables are listed in Table 1. With regard to changes in job satisfaction, three-fourths of the respondents answered, “Unchanged”, while almost one-fifth answered, “Deteriorated/Heavily deteriorated”. Regarding the change in satisfaction with family, about four-fifths answered, “Unchanged”, while almost one-tenth answered, “Deteriorated/Heavily deteriorated” or “Improved/Heavily Improved”, respectively. Meanwhile, regarding the change in psychological well-being, about half of the total respondents answered, “Unchanged”, while almost two-fifths answered, “Deteriorated/Heavily deteriorated”. Furthermore, regarding the change in economic well-being, almost two-thirds answered, “Unchanged”, while almost everyone else answered, “Deteriorated/Heavily deteriorated”.

Based on these results, it appears that both psychological and economic well-being has deteriorated more than job satisfaction and satisfaction with family. In addition, the proportion of Improved/Heavily Improved for change in satisfaction with family is almost the same as that of Deteriorated/Heavily deteriorated; thus, it seems to imply that COVID-19 may influence satisfaction with family, both positively and negatively.

### 3.2. OLR

#### 3.2.1. Change in Job Satisfaction

The results of the OLR, whose dependent variable is change in job satisfaction, are shown in Table 2. For model fitting, the chi-square test for -2 log-likelihood (-2LL) values of the intercept-only model and the final model indicates statistical significance (*p* = 0.003) at the 5% level, which means that the final model has significant improvement over the intercept-only model. All thresholds are statistically significant, while two independent variables, namely, change in daily food, water, electricity, and heat consumption and family structure, show statistical significance. Notably, positive coefficients lead to a decrease in cumulative logit and vice versa in SPSS OLR, and we find that the more daily food, water, electricity, and heat consumption increase, the more job satisfaction deteriorates. Similarly, the job satisfaction of people who do not belong to a single-person household appears to have improved. This seems to be because remote work has become more popular owing to the COVID-19 pandemic. It costs more in daily food, water, electricity, and heat consumption to perform remote work, which may lead to a decrease in job satisfaction. Meanwhile, remote working can provide more time to stay with families. Therefore, it is possible that people who do not have a single-person household are satisfied with their jobs because of the introduction of remote work.

#### 3.2.2. Change in Satisfaction with Family

The results of the OLR, whose dependent variable is change in satisfaction with family, are shown in Table 3. The chi-square test for -2LL values indicates statistical significance (*p* = 0.019) at the 5% level. Thresholds other than Deteriorated are statistically significant, while four independent variables, namely, concerns about the possibility of simultaneous occurrence of natural hazards, family structure, length of residency, and the existence of dependents, show statistical significance at the 5% level. This may imply that satisfaction with a family of people who are concerned about the possibility of a simultaneous occurrence of disasters caused by natural hazards, tends to deteriorate during the COVID-19 pandemic. We also found that satisfaction with a family of people, who do not belong to a single-person household, seems to have improved as well as job satisfaction, while the short length of residency (less than 10 years) and the inexistence of dependents appear to have a positive impact on change in satisfaction with family during the COVID-19 pandemic era. Notably, a latent variable may exist behind these independent variables, and further study is needed to better understand the results.

#### 3.2.3. Change in Psychological Well-Being

The results of the OLR, whose dependent variable is change in psychological well-being, are shown in Table 4. The chi-square test for -2LL values indicates statistical significance (*p* < 0.001) at the 5% level. Thresholds other than Deteriorated are statistically significant, while six independent variables, namely, change in use of private transportation, change in the use of telephone and Internet services, education level, family structure, the existence of dependents, and residency in the Greater Tokyo Area, show statistical significance at the 5% level. It seems that an increase in the use of private transportation has a positive impact on psychological well-being, while an increase in the use of telephone and Internet services, which may be caused by remote work due to the COVID-19 pandemic, had an adverse impact. It also appears that the psychological well-being of people, whose education level is not at university graduation or above, has deteriorated and that of people, who do not belong to a single-person household, has a tendency to improve. Furthermore, the existence of dependents appears to have a positive impact on change in psychological well-being, while residency in a place other than the Greater Tokyo area seems to have a negative impact.

#### 3.2.4. Change in Economic Well-Being

The results of the OLR, whose dependent variable is change in economic well-being, are shown in Table 5. The chi-square test for -2LL values indicates statistical significance (*p* < 0.001) at the 5% level. All thresholds are statistically significant, while five independent variables, namely, change in daily food, water, electricity, and heat consumption, concerns about the lack of economic recovery measures, concerns about possible disruption of essential and basic services, education level, and family structure, show statistical significance at the 5% level. It seems plausible that increases in daily food, water, electricity, and heat consumption that may be caused by remote work, as well as concerns about the lack of economic recovery measures and concerns about possible disruption of essential and basic services, have a negative impact on economic well-being because these appear to have a straightforward relationship. In addition, the economic well-being, as well as psychological well-being, of people, whose education level is not at university graduation or above, seems to have deteriorated and that of people who do not belong to a single-person household appeared to have a tendency to improve, as observed for the other three dependent variables.

### 3.3. Creation of a New CI

#### 3.3.1. PCA

As mentioned in the Methodology section, the four variables of people’s perception of well-being under the COVID-19 pandemic, namely, change in job satisfaction, change in satisfaction with family, change in psychological well-being, and change in economic well-being, are input; furthermore, we assume that they range from −2 (Heavily deteriorated) to 2 (Heavily improved). The total explained variance is presented in Table 6. Based on this, we can find that only one principal component was to be extracted by the Kaiser–Guttman criterion; that is, components whose eigenvalues exceed 1 should be extracted, and 51% of the total variance is explained by PC1. The component matrix, as shown in Table 7, implies that changes in psychological well-being and changes in economic well-being may be slightly more correlated with PC1 than changes in job satisfaction and change in satisfaction with family.

**Figure 1:**
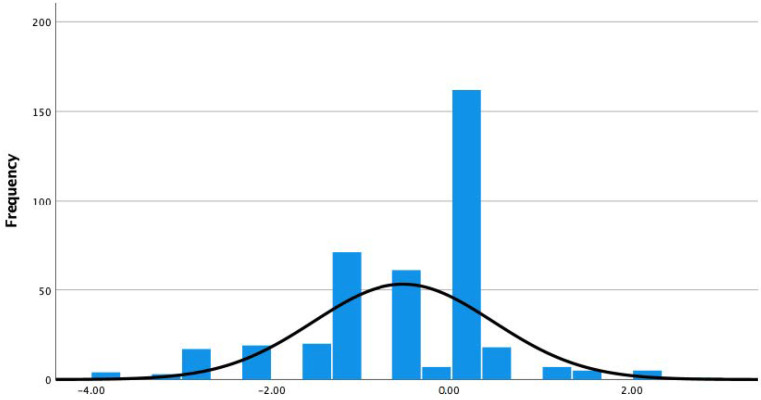
Histogram of the CI.

Subsequently, the authors calculated the PCS of PC1, namely a new CI, while adjusting its values so that the CI became zero (Unchanged) when all four original input variables have the value of zero (Unchanged). The descriptive statistics and histograms of the CI are shown in Table 8 and Figure 1. We find that the distribution of the CI is skewed toward the negative side, although many are distributed near zero (unchanged).

#### 3.3.2. OLS Regression

**Figure 2:**
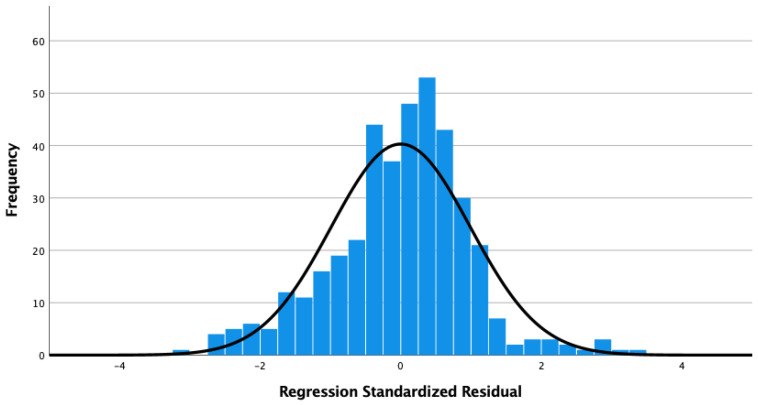
Histogram of the regression standardized residual.

We conducted an OLS regression with a forward-backward stepwise selection method, whose dependent variable was the CI, while the independent variables were the same as in the abovementioned OLR. The coefficients of the final selected model and the histogram of standardized residuals are shown in Table 9 and Figure 2. The result of the analysis of variance (ANOVA) is significant (*p* < 0.001), and the adjusted R-square is 0.143. All variance inflation factors (VIF) are less than 10.0, which implies no multicollinearity. The Durbin–Watson ratio is 1.842. It is generally acceptable to assume that the residuals are normally distributed.

## 4. Discussion

Based on the results of OLS, eight independent variables, namely changes in daily food, water, electricity, and heat consumption, concerns about the risk of a new wave of COVID-19 infection spreading, concerns about possible disruption of essential and basic services, education level, age, family structure, the existence of dependents, and annual household income, show statistical significance at the 5% level. Three out of eight independent variables, namely concerns about the risk of a new wave of COVID-19 infection spreading, age, and annual household income, do not show statistical significance in either case of OLR. Meanwhile, the family structure shows statistical significance in all cases of OLR. In terms of standardized coefficients (Beta), it seems that family structure has the greatest impact on CI, which is assumed to represent people’s perception of well-being under the COVID-19 pandemic in general terms, followed by education level.

It should also be noted that there seems to be a non-significant difference between genders in neither case of OLR/OLS. Meanwhile, Nienhuis and Lesser [11] stated that the analysis based on the data provided by 1098 Canadians, 215 men and 871 women, showed sex differences in physical activity and well-being. Considering that approaches to family for males and females in Japan are different, this result has implications.

In general, it is imperative to prioritize policy targets due to time and budget constraints. As a result of this study, we can assert with evidence that policies for single-person households would improve their well-being effectively and efficiently. This argument seems to be unfamiliar in Japan at the moment, and thus, it is worth reconsidering how the government should allocate limited policy resources to address the ongoing pandemic.

## 5. Conclusions

In this study, we quantitatively examined people’s perceptions of well-being during the COVID-19 pandemic in Japan. In the OLR analysis, some independent variables, which were not common but specific for each dependent variable, demonstrated statistical significance. Meanwhile, the CI created by utilizing PCA in this study provides an option to grasp people’s perceptions of well-being. As discussed above, eight independent variables, namely, change in daily food, water, electricity, and heat consumption, concerns about the risk of a new wave of COVID-19 infection spreading, concerns about the possible disruption of essential and basic services, education level, age, family structure, the existence of dependents, and annual household income, are statistically significant at the 5% level in the OLS analysis, whose dependent variable is the CI. Furthermore, we found that family structure had the greatest impact on CI, which was consistent with the results of the OLR analysis. Therefore, we can identify the family structure as a key factor in the realization of EBPM in the future.

Based on the results of this study, the authors advocate that the Japanese government should pay more attention to single-person households affected by the COVID-19 pandemic. Some policies regarding COVID-19 in Japan seemingly tend to be implemented for households consisting of more than one person, such as households with children. The literature review in the Introduction section also indicates that it may be of great significance to address loneliness in the COVID-19 era. We hope that our study can also contribute to the provision of evidence for future policymaking for single-person households in Japan.

The future research focus should be two-fold: (i) to expand to research areas outside Japan so that we can compare results in a cross-sectional manner and verify the validity of the CI created in this study and (ii) to acquire time series data in Japan to assess Japanese policies regarding the COVID-19 pandemic. This cross-sectional and time series analysis could establish a comprehensive and exhaustive framework for evaluating people’s perception of well-being during the COVID-19 pandemic and assess relevant policies in a quantitative manner, thus, contributing to the literature on EBPM in the field of disaster science.

## Figures and Tables

**Table 1:** Frequency distribution of the dependent variables.

	Total *n* (%)	Heavily Deteriorated *n* (%)	Deteriorated *n* (%)	Unchanged *n* (%)	Improved *n* (%)	Heavily Improved *n* (%)
Change in job satisfaction	400	19	63	300	17	1
	(100.0)	(4.8)	(15.8)	(75.0)	(4.3)	(0.3)
Change in satisfaction with family	400	4	40	314	36	6
	(100.0)	(1.0)	(10.0)	(78.5)	(9.0)	(1.5)
Change in psychological well-being	400	31	128	216	22	3
	(100.0)	(7.8)	(32.0)	(54.0)	(5.5)	(0.8)
Change in economic well-being	400	32	101	256	11	0
	(100.0)	(8.0)	(25.3)	(64.0)	(2.8)	(0.0)

**Table 2:** Parameter estimates for change in job satisfaction.

		Estimate	SE	Wald	df	Sig.	95% CI	
							Lower	Upper
**Threshold**	**Heavily deteriorated**	**−4.578**	**0.952**	**23.112**	**1**	**<0.001**	**−6.445**	**−2.712**
	**Deteriorated**	**−2.771**	**0.924**	**8.995**	**1**	**0.003**	**−4.582**	**−0.960**
	**Unchanged**	**2.052**	**0.921**	**4.962**	**1**	**0.026**	**0.246**	**3.857**
	**Improved**	**4.975**	**1.329**	**14.017**	**1**	**<0.001**	**2.371**	**7.580**
**Location**	**Change in daily food, water, electricity and heat consumption**	**−0.428**	**0.213**	**4.060**	**1**	**0.044**	**−0.845**	**−0.012**
	Change in use of public transportation	0.093	0.231	0.162	1	0.688	−0.360	0.545
	Change in use of private transportation	0.101	0.242	0.175	1	0.675	−0.372	0.575
	Change in use of medical and hospital services	−0.052	0.213	0.060	1	0.807	−0.469	0.365
	Change in use of banking and financial services	0.075	0.264	0.080	1	0.777	−0.443	0.592
	Change in use of telephone and internet services	−0.035	0.202	0.029	1	0.864	−0.430	0.361
	Concerns about the lack of economic recovery measures	−0.056	0.122	0.213	1	0.644	−0.296	0.183
	Concerns about the risk of a new wave of COVID-19 infection spreading	−0.199	0.130	2.351	1	0.125	−0.453	0.055
	Concerns about the possible disruption of essential and basic services	−0.013	0.161	0.006	1	0.936	−0.328	0.302
	Concerns about the possibility of simultaneous occurrence of natural hazards	−0.226	0.161	1.975	1	0.160	−0.541	0.089
	Concerns about the risk of simultaneous acts of terrorism, cyber-attacks, riots	0.028	0.151	0.035	1	0.851	−0.267	0.323
	Age	−0.018	0.014	1.685	1	0.194	−0.044	0.009
	Number of households	−0.008	0.024	0.107	1	0.744	−0.054	0.039
	[Gender = 0]	−0.327	0.273	1.434	1	0.231	−0.862	0.208
	[Education level = 0]	−0.194	0.247	0.615	1	0.433	−0.677	0.290
	**[Family structure = 0]**	**0.967**	**0.318**	**9.252**	**1**	**0.002**	**0.344**	**1.590**
	[Length of residency = 0]	−0.061	0.262	0.053	1	0.817	−0.574	0.453
	[Existence of dependents = 0]	0.297	0.275	1.164	1	0.281	−0.243	0.837
	[Existence of pets = 0]	0.105	0.297	0.125	1	0.723	−0.477	0.687
	[Employment = 0]	0.276	0.278	0.987	1	0.321	−0.268	0.820
	[Annual household income = 0]	0.178	0.258	0.475	1	0.491	−0.328	0.684
	[Residency in the Greater Tokyo area = 0]	−0.202	0.291	0.482	1	0.488	−0.772	0.368
Pseudo R-square								
Cox and Snell		0.105						
Nagelkerke		0.131						
McFadden		0.069						

Bold font indicates statistical significance at the 5% level.

**Table 3:** Parameter estimates for change in satisfaction with family.

		Estimate	SE	Wald	df	Sig.	95% CI	
							Lower	Upper
**Threshold**	**Heavily deteriorated**	**−3.412**	**1.051**	**10.534**	**1**	**0.001**	**−5.473**	**−1.352**
	Deteriorated	−0.864	0.942	0.841	1	0.359	−2.710	0.983
	**Unchanged**	**3.815**	**0.974**	**15.345**	**1**	**<0.001**	**1.906**	**5.723**
	**Improved**	**5.941**	**1.050**	**32.016**	**1**	**<0.001**	**3.883**	**7.999**
**Location**	Change in daily food, water, electricity and heat consumption	0.205	0.229	0.807	1	0.369	−0.243	0.654
	Change in use of public transportation	−0.142	0.237	0.359	1	0.549	−0.607	0.323
	Change in use of private transportation	0.149	0.252	0.352	1	0.553	−0.344	0.643
	Change in use of medical and hospital services	−0.172	0.230	0.559	1	0.455	−0.624	0.279
	Change in use of banking and financial services	−0.066	0.291	0.051	1	0.821	−0.636	0.505
	Change in use of telephone and internet services	0.191	0.216	0.783	1	0.376	−0.233	0.616
	Concerns about the lack of economic recovery measures	0.015	0.129	0.013	1	0.909	−0.238	0.268
	Concerns about the risk of a new wave of COVID-19 infection spreading	0.084	0.133	0.397	1	0.528	−0.177	0.344
	Concerns about the possible disruption of essential and basic services	0.145	0.171	0.723	1	0.395	−0.190	0.481
	**Concerns about the possibility of simultaneous occurrence of natural hazards**	**−0.356**	**0.170**	**4.403**	**1**	**0.036**	**−0.688**	**−0.023**
	Concerns about the risk of simultaneous acts of terrorism, cyber-attacks, riots	0.047	0.160	0.087	1	0.767	−0.265	0.360
	Age	−0.007	0.014	0.212	1	0.645	−0.034	0.021
	Number of households	0.035	0.026	1.801	1	0.180	−0.016	0.087
	[Gender = 0]	0.395	0.289	1.876	1	0.171	−0.170	0.961
	[Education level = 0]	−0.272	0.262	1.075	1	0.300	−0.786	0.242
	**[Family structure = 0]**	**1.148**	**0.351**	**10.706**	**1**	**0.001**	**0.460**	**1.835**
	**[Length of residency = 0]**	**0.558**	**0.276**	**4.099**	**1**	**0.043**	**0.018**	**1.098**
	**[Existence of dependents = 0]**	**0.574**	**0.289**	**3.942**	**1**	**0.047**	**0.007**	**1.141**
	[Existence of pets = 0]	0.357	0.314	1.297	1	0.255	−0.258	0.972
	[Employment = 0]	−0.243	0.292	0.693	1	0.405	−0.816	0.329
	[Annual household income = 0]	0.090	0.274	0.107	1	0.743	−0.448	0.627
	[Residency in the Greater Tokyo area = 0]	−0.442	0.307	2.078	1	0.149	−1.043	0.159
Pseudo R-square								
Cox and Snell		0.090						
Nagelkerke		0.117						
McFadden		0.063						

Bold font indicates statistical significance at the 5% level.

**Table 4:** Parameter estimates for change in psychological well-being.

		Estimate	SE	Wald	df	Sig.	95% CI	
							Lower	Upper
**Threshold**	**Heavily deteriorated**	**−3.640**	**0.793**	**21.054**	**1**	**<0.001**	**−5.194**	**−2.085**
	Deteriorated	−1.283	0.770	2.777	1	0.096	−2.791	0.226
	**Unchanged**	**2.204**	**0.781**	**7.971**	**1**	**0.005**	**0.674**	**3.734**
	**Improved**	**4.398**	**0.946**	**21.602**	**1**	**<0.001**	**2.543**	**6.252**
**Location**	Change in daily food, water, electricity and heat consumption	−0.247	0.184	1.796	1	0.180	−0.608	0.114
	Change in use of public transportation	−0.302	0.199	2.292	1	0.130	−0.693	0.089
	**Change in use of private transportation**	**0.445**	**0.210**	**4.520**	**1**	**0.034**	**0.035**	**0.856**
	Change in use of medical and hospital services	0.328	0.185	3.144	1	0.076	−0.035	0.690
	Change in use of banking and financial services	0.129	0.234	0.307	1	0.580	−0.328	0.587
	**Change in use of telephone and internet services**	**−0.346**	**0.174**	**3.961**	**1**	**0.047**	**−0.687**	**−0.005**
	Concerns about the lack of economic recovery measures	−0.072	0.104	0.477	1	0.490	−0.277	0.132
	Concerns about the risk of a new wave of COVID-19 infection spreading	−0.182	0.109	2.760	1	0.097	−0.396	0.033
	Concerns about the possible disruption of essential and basic services	−0.065	0.140	0.215	1	0.643	−0.338	0.209
	Concerns about the possibility of simultaneous occurrence of natural hazards	0.036	0.138	0.067	1	0.796	−0.236	0.307
	Concerns about the risk of simultaneous acts of terrorism, cyber-attacks, riots	−0.031	0.131	0.057	1	0.811	−0.289	0.226
	Age	−0.012	0.012	1.017	1	0.313	−0.034	0.011
	Number of households	0.035	0.023	2.291	1	0.130	−0.010	0.081
	[Gender = 0]	0.008	0.232	0.001	1	0.974	−0.447	0.463
	**[Education level = 0]**	**−0.529**	**0.211**	**6.271**	**1**	**0.012**	**−0.943**	**−0.115**
	**[Family structure = 0]**	**1.005**	**0.280**	**12.842**	**1**	**<0.001**	**0.455**	**1.554**
	[Length of residency = 0]	0.354	0.223	2.518	1	0.113	−0.083	0.792
	**[Existence of dependents = 0]**	**0.556**	**0.233**	**5.700**	**1**	**0.017**	**0.100**	**1.013**
	[Existence of pets = 0]	−0.138	0.254	0.297	1	0.586	−0.636	0.359
	[Employment = 0]	−0.182	0.234	0.604	1	0.437	−0.642	0.277
	[Annual household income = 0]	0.233	0.220	1.120	1	0.290	−0.198	0.664
	**[Residency in the Greater Tokyo area = 0]**	**−0.563**	**0.253**	**4.971**	**1**	**0.026**	**−1.058**	**−0.068**
Pseudo R-square								
Cox and Snell		0.165						
Nagelkerke		0.186						
McFadden		0.083						

Bold font indicates statistical significance at the 5% level.

**Table 5:** Parameter estimates for change in economic well-being.

		Estimate	SE	Wald	df	Sig.	95% CI	
							Lower	Upper
**Threshold**	**Heavily deteriorated**	**−4.633**	**0.871**	**28.281**	**1**	**<0.001**	**−6.341**	**−2.926**
	**Deteriorated**	**−2.578**	**0.845**	**9.304**	**1**	**0.002**	**−4.234**	**−0.921**
	**Unchanged**	**2.218**	**0.862**	**6.625**	**1**	**0.010**	**0.529**	**3.906**
**Location**	**Change in daily food, water, electricity and heat consumption**	**−0.492**	**0.197**	**6.238**	**1**	**0.013**	**−0.878**	**−0.106**
	Change in use of public transportation	−0.002	0.216	0.000	1	0.993	−0.425	0.421
	Change in use of private transportation	0.241	0.223	1.171	1	0.279	−0.196	0.678
	Change in use of medical and hospital services	0.192	0.195	0.966	1	0.326	−0.191	0.575
	Change in use of banking and financial services	−0.143	0.248	0.331	1	0.565	−0.629	0.344
	Change in use of telephone and internet services	0.019	0.185	0.010	1	0.920	−0.344	0.382
	**Concerns about the lack of economic recovery measures**	**−0.222**	**0.112**	**3.921**	**1**	**0.048**	**−0.441**	**−0.002**
	Concerns about the risk of a new wave of COVID-19 infection spreading	−0.174	0.117	2.203	1	0.138	−0.405	0.056
	**Concerns about the possible disruption of essential and basic services**	**−0.316**	**0.149**	**4.510**	**1**	**0.034**	**−0.607**	**−0.024**
	Concerns about the possibility of simultaneous occurrence of natural hazards	0.290	0.151	3.705	1	0.054	−0.005	0.586
	Concerns about the risk of simultaneous acts of terrorism, cyber-attacks, riots	−0.171	0.139	1.515	1	0.218	−0.443	0.101
	Age	−0.018	0.012	2.170	1	0.141	−0.043	0.006
	Number of households	0.025	0.025	0.972	1	0.324	−0.025	0.075
	[Gender = 0]	−0.106	0.250	0.180	1	0.671	−0.596	0.384
	**[Education level = 0]**	**−0.617**	**0.228**	**7.297**	**1**	**0.007**	**−1.065**	**−0.169**
	**[Family structure = 0]**	**0.599**	**0.297**	**4.072**	**1**	**0.044**	**0.017**	**1.181**
	[Length of residency = 0]	0.130	0.240	0.295	1	0.587	−0.340	0.601
	[Existence of dependents = 0]	0.204	0.250	0.664	1	0.415	−0.286	0.694
	[Existence of pets = 0]	0.310	0.267	1.356	1	0.244	−0.212	0.833
	[Employment = 0]	−0.391	0.250	2.432	1	0.119	−0.881	0.100
	[Annual household income = 0]	0.440	0.235	3.500	1	0.061	−0.021	0.901
	[Residency in the Greater Tokyo area = 0]	−0.378	0.274	1.904	1	0.168	−0.915	0.159
Pseudo R-square								
Cox and Snell		0.183						
Nagelkerke		0.216						
McFadden		0.108						

Bold font indicates statistical significance at the 5% level.

**Table 6:** Total variance explained of PCA.

Component	Initial Eigenvalues			Extraction Sums of Squared Loadings		
	Total	% of Variance	Cumulative %	Total	% of Variance	Cumulative %
1	2.057	51.422	51.422	2.057	51.422	51.422
2	0.883	22.066	73.488			
3	0.598	14.942	88.430			
4	0.463	11.570	100.000			
Extraction Method: Principal Component Analysis						

**Table 7:** Component matrix of PCA.

	Component 1
change in psychological well-being	0.815
change in economic well-being	0.753
change in job satisfaction	0.678
change in satisfaction with family	0.605
Extraction Method: Principal Component Analysis	

**Table 8:** Descriptive statistics of the CI.

	CI
Mean	−0.5382
Median	−0.0047
Std. Deviation	1.00000
Variance	1.000
Skewness	−0.577
Std. Error of Skewness	0.122
Kurtosis	1.407
Std. Error of Kurtosis	0.243
Minimum	−3.80
Maximum	2.72

**Table 9:** Coefficients of the finally selected model.

	Unstandardized Coefficients		Standardized Coefficients	t	Sig.	95% Confidence Interval for B	
	B	Std. Error	Beta			Lower	Upper
(Constant)	0.559	0.264		2.118	0.035	0.040	1.078
Change in daily food, water, electricity and heat consumption	−0.155	0.076	−0.100	−2.044	0.042	−0.305	−0.006
Concerns about the risk of a new wave of COVID-19 infection spreading	−0.098	0.046	−0.111	−2.117	0.035	−0.189	−0.007
Concerns about the possible disruption of essential and basic services	−0.112	0.049	−0.119	−2.276	0.023	−0.210	−0.015
Education level	0.274	0.094	0.137	2.902	0.004	0.088	0.460
Age	−0.011	0.005	−0.111	−2.348	0.019	−0.020	−0.002
Family structure	−0.534	0.124	−0.221	−4.301	<0.001	−0.779	−0.290
Existence of dependents	−0.226	0.104	−0.107	−2.163	0.031	−0.431	−0.021
Annual household income	−0.210	0.100	−0.103	−2.097	0.037	−0.406	−0.013

## Data Availability

The data presented in this study are available upon request.

## References

[1] Saengtabtim K., Leelawat N., Tang J., Suppasri A., Imamura F. (2022). Consequences of COVID-19 on health, economy, and tourism in Asia: A systematic review. Sustainability.

[2] Holmes E., O’Connor R., Perry V., Tracey I., Wessely S., Arseneault L., Ballard C., Christensen H., Silver R.C., Everall I. (2020). Multidisciplinary research priorities for the COVID-19 pandemic: A call for action for mental health science. Lancet Psychiatry.

[3] Chatterjee R., Bajwa S., Dwivedi D., Kanji R., Ahammed M., Shaw R. (2020). COVID-19 Risk Assessment Tool: Dual application of risk communication and risk governance. Prog. Disaster Sci..

[4] Djalante R., Shaw R., DeWit A. (2020). Building resilience against biological hazards and pandemics: COVID-19 and its implications for the Sendai Framework. Prog. Disaster Sci..

[5] Shaw R., Kim Y., Hua J. (2020). Governance, technology and citizen behavior in pandemic: Lessons from COVID-19 in East Asia. Prog. Disaster Sci..

[6] Aknin L., De Neve J., Dunn E., Fancourt D., Goldberg E., Helliwell J.F., Jones S.P., Karam E., Layard R., Lyubomirsky S. (2022). Mental Health During the First Year of the COVID-19 Pandemic: A Review and Recommendations for Moving Forward. Perspect. Psychol. Sci..

[7] Saladino V., Algeri D., Auriemma V. (2020). The psychological and social impact of Covid-19: New perspectives of well-being. Front. Psychol..

[8] Sibley C.G., Greaves L.M., Satherley N., Wilson M.S., Overall N.C., Lee C.H.J., Milojev P., Bulbulia J., Osborne D., Milfont T.L. (2020). Effects of the COVID-19 pandemic and nationwide lockdown on trust, attitudes toward government, and well-being. Am. Psychol..

[9] Brodeur A., Clark A.E., Fleche S., Powdthavee N. (2021). COVID-19, lockdowns and well-being: Evidence from Google Trends. J. Public Econ..

[10] Lesser I.A., Nienhuis C.P. (2020). The impact of COVID-19 on physical activity behavior and well-being of Canadians. Int. J. Environ. Res. Public Health.

[11] Nienhuis C.P., Lesser I.A. (2020). The impact of COVID-19 on women’s physical activity behavior and mental well-being. Int J Env. Res Public Health.

[12] Dahlen M., Thorbjørnsen H., Sjåstad H., von Heideken Wågert P., Hellström C., Kerstis B., Lindberg D., Stier J., Elvén M. (2021). Changes in physical activity are associated with corresponding changes in psychological well-being: A pandemic case study. Int. J. Environ. Res. Public Health.

[13] Feitelson E., Plaut P., Salzberger E., Shmueli D., Altshuler A., Ben-Gal M., Israel F., Rein-Sapir Y., Zaychik D. (2022). The effects of COVID-19 on wellbeing: Evidence from Israel. Sustainability.

[14] Gassman-Pines A., Ananat E.O., Fitz-Henley J. (2020). COVID-19 and parent-child psychological well-being. Pediatrics.

[15] Patrick S.W., Henkhaus L.E., Zickafoose J.S., Lovell K., Halvorson A., Loch S., Letterie M., Davis M.M. (2020). Well-being of parents and children during the COVID-19 pandemic: A national survey. Pediatrics.

[16] Huebener M., Waights S., Spiess C.K., Siegel N.A., Wagner G.G. (2021). Parental well-being in times of Covid-19 in Germany. Rev. Econ. Househ..

[17] O’Connor R., Wetherall K., Cleare S., McClelland H., Melson A., Niedzwiedz C.L., O’Carroll R.E., O’Connor D.B., Platt S., Scowcroft E. (2020). Mental health and well-being during the COVID-19 pandemic: Longitudinal analyses of adults in the UK COVID-19 Mental Health & wellbeing study. Br. J. Psychiatry.

[18] Coppola I., Rania N., Parisi R., Lagomarsino F. (2021). Spiritual well-being and mental health during the COVID-19 pandemic in Italy. Front. Psychiatry.

[19] Özmen S., Özkan O., Özer Ö., Yanardağ M.Z. (2021). Investigation of COVID-19 fear, well-being and life satisfaction in Turkish society. Soc. Work Public Health.

[20] Tomaz S.A., Coffee P., Ryde G.C., Swales B., Neely K.C., Connelly J., Kirkland A., McCabe L., Watchman K., Andreis F. (2021). Loneliness, wellbeing, and social activity in Scottish older adults resulting from social distancing during the COVID-19 pandemic. Int. J. Environ. Res. Public Health.

[21] Fingerman K., Ng Y., Zhang S., Britt K., Colera G., Birditt K., Charles S. (2020). Living alone during COVID-19: Social contact and emotional well-being among older adults. J. Gerontol. Ser. B.

[22] Egawa S., Jibiki Y., Sasaki D., Ono Y., Nakamura Y., Suda T., Sasaki H. (2018). The correlation between life expectancy and disaster risk. J. Disaster. Res..

[23] Moriyama K., Sasaki D., Ono Y. (2018). Comparison of global databases for disaster loss and damage data. J. Disaster. Res..

[24] Sasaki D., Moriyama K., Ono Y. (2018). Hidden common factors in disaster loss statistics: A case study analyzing the data of Nepal. J. Disaster. Res..

[25] Sasaki D., Ono Y. (2018). Overview of the special issue on the development of disaster statistics. J. Disaster. Res..

[26] Sakamoto M., Sasaki D., Ono Y., Makino Y., Kodama E.N. (2020). Implementation of evacuation measures during natural disasters under conditions of the novel coronavirus (COVID-19) pandemic based on a review of previous responses to complex disasters in Japan. Prog. Disaster. Sci..

[27] Sasaki D., Moriyama K., Ono Y. (2020). Main features of the existing literature concerning disaster statistics. Int. J. Disaster. Risk Reduc..

[28] Suppasri A., Kitamura M., Tsukuda H., Boret S.P., Pescaroli G., Onoda Y., Imamura F., Alexander D., Leelawat N., Syamsidik (2021). Perceptions of the COVID-19 pandemic in Japan with respect to cultural, information, disaster and social issues. Prog. Disaster. Sci..

[29] Pescaroli G., Galbusera L., Cardarilli M., Giannopoulos G., Alexander D. (2021). Linking healthcare and societal resilience during the Covid-19 pandemic. Saf. Sci..

[30] DeMaris A. (1995). A tutorial in logistic regression. J. Marriage Fam..

[31] Cabinet Office (CAO) (2022). Activities Regarding Well-Being.

[32] Hotelling H. (1933). Analysis of a complex of statistical variables into principal components. J. Educ. Psychol..

[33] Jolliffe I.T., Cadima J. (2016). Principal component analysis: A review and recent developments. Philos. Trans. A Math. Phys. Eng. Sci..

